# Super-Recognizers, or Su-Perceivers? Insights from fast periodic visual stimulation (FPVS) EEG

**DOI:** 10.1007/s10548-025-01136-9

**Published:** 2025-08-28

**Authors:** Jeffrey D. Nador, Kim Uittenhove, Dario Gordillo, Meike Ramon

**Affiliations:** 1 Applied Face Cognition Lab, Business School, Bern University of Applied Sciences, Bern, Switzerland; 2 Center for Learning Science, EPFL, Lausanne, Switzerland; 3 Laboratory of Psychophysics, École polytechnique fédérale de Lausanne, Lausanne, Switzerland; 4 AIR – Association for Independent Research, Zurich, Switzerland

**Keywords:** Super-Recognizers, Neurofunctioning, FPVS EEG, Face perception and recognition, Policing

## Abstract

The term *Super-Recognizer (SR)*, which describes individuals with supposedly superior facial recognition abilities, may be something of a misnomer. In the same way that blind individuals would not be considered prosopagnosic, SR diagnoses should emphasise at least face identity processing (FIP) specificity, if not recognition in particular. However, SRs tend to be diagnosed with face-specific *behavioral* tasks, probing either perception *and/or* recognition, and leaving the neural basis and mechanisms underlying their abilities largely unexplored. The present study therefore sought to determine whether any common FIP subprocesses, among a sample of stringently and comparably diagnosed SRs, would distinguish them from neurotypical controls. To this end, we conducted three Fast Periodic Visual Stimulation (FPVS) EEG experiments in a group of Berlin Police officers identified as SRs using the only existing formal diagnostic framework for lab-based SR identification (Ramon in Neuropsychologia 158:107809, https://doi.org/10.1016/j.neuropsychologia.2021.107809, 2021) that aligns with the seminal study of SRs (Russell et al. in Psychon Bull Rev 16(2):252–257, https://doi.org/10.3758/PBR.16.2.252, 2009). These experiments aimed to isolate FIP from behavioral and general perceptual factors in terms of both the consistency and speed of face identity discrimination and categorization. Broadly, the results of all three experiments provided two key findings. First, whichever factors distinguish SRs from controls, they are not face-specific. Second, SRs are not all cut from the same cloth. Rather, the factors distinguishing SRs from controls seem to be individual-specific, warranting more nuanced and bespoke testing criteria for their deployment in practical applications.

## Introduction

Face identity processing (FIP)––while seemingly trivial in daily life––requires a number of processing steps to unfold successfully (Liu et al. 2002; Yovel et al. 2003; Tsao and Livingstone 2008; Ramon and Gobbini 2018; Ramon and Rjosk 2022; Besson et al. 2017). Broadly speaking, perceptual processing precedes face recognition and culminates in identification. Visual stimuli are initially processed categorically, enabling rapid face *detection* (Crouzet et al. 2010; Crouzet and Thorpe 2011). An established facial representation can then serve perceptual *discrimination*, (exposure-dependent) memory-based *recognition,* and (provided prior familiarity and semantic knowledge) *identification* of an individual’s unique identity.

### Individual differences across the FIP ability spectrum

These FIP sub-processes may be impaired in acquired prosopagnosia, the selective deficit affecting FIP caused by neurological damage (for reviews see e.g., Rossion 2014, 2018). While prosopagnosic patients’ impairments have traditionally been classified as perceptual or mnemonic (Barton et al. 2021; Anaki et al. 2007; De Renzi et al. 1991; Davies-Thompson et al. 2014), closer inspection has revealed that impaired perceptual processing is the underlying common feature across varied lesion locations (Busigny et al. 2010a, b; Delvenne et al. 2004; Busigny et al. 2014; Ramon et al. 2010).

In contrast to the rare, yet heavily studied, phenomenon of prosopagnosia, it is only relatively recently that individuals with markedly *superior* FIP skills have become the subject of investigation. These accidentally discovered ‘Super-Recognizers’ (SRs) are “about as good at face recognition and perception as developmental prosopagnosics are bad” (Russell et al. 2009; p. 252). Since their discovery, development and elaboration of diagnostic criteria (Ramon, 2021; Bate et al. 2019) for translational applications in personnel selection (e.g. Ramon et al. 2019a, b; Nador et al. 2022; Ramon and Vowels 2025; Ramon and Rjosk 2022; Ramon et al. 2024) have become the predominant focus of research on these rare individuals. For instance, law enforcement and police-practitioners are increasingly seeking to exploit their abilities, with a view to improving their operations (Nador et al. 2022; Ramon and Rjosk 2021; Mayer and Ramon 2023; Ramon et al. 2019a, b). However, selection criteria employed in these contexts largely overlook inter- and intra-individual variability in their FIP abilities (e.g., Abudarham et al. 2021; Robertson et al. 2016; Dunn et al. 2020; Abudarham et al. 2021; Mayer and Ramon 2023; but see Bobak et al. 2016; Faghel-Soubeyrand et al. 2024).

### Limited insights into “Super-Recognizers” neuro–functioning

Mirroring the recent surge in interest in individual differences in neurofunctioning more broadly, and FIP specifically (Bobak et al. 2023; Stacchi et al. 2020; Fysh et al. 2020), the literature into SRs is growing. Overall, however, two main issues persist. First, most SR studies either do not distinguish between different FIP sub-processes (e.g., Phillips et al. 2018; Dunn et al. 2020; for a discussion see Ramon 2021; Ramon and Vowels, 2025), and/or focus only on face recognition (Bate et al. 2021). Such approaches are thus at odds with the seminal behavioral study that clearly reported superior FIP across a range of possible sub-processes (Russell et al. 2009).

For this reason, over the past years, the Applied Face Cognition Lab has directed concerted efforts towards the systematic investigation of factors that determine SRs’ performance across different FIP subprocesses using various behavioral methods (Nador et al. 2021a, b, 2022; Linka et al. 2022; Mayer & Ramon, 2023; Marini et al. 2024; for a recent review see Ramon, 2023). Collectively, the findings suggest that SRs’ processing advantages (whether face-specific or not) are heterogeneous across tasks, and depending on the FIP sub-processes they involve (Bobak et al. 2016; Ramon 2021; Nador et al. 2021a, b; Ramon and Rjosk 2022; Ramon and Vowels, 2025).

Second, to our knowledge, only two studies have reported electrophysiological data from individuals reported as SRs (identified as such based on CFMT + data alone), acquired during concurrent stimulus-directed, *memory* tasks. In the context of an old/new “Adult and Infant Face Recognition Test”, Belanova et al. (2018; Experiment 2), reported enhanced P1 and P600 amplitudes for SRs compared to controls, but no differences for N170 or N250 components. Using a 1*-*back task with varied non-/face stimulus categories, Faghel-Soubeyrand et al. (2024) reported reliable, and stimulus category independent classification of observers’ group status from 70 ms post stimulus onset onwards.

SR diagnostic shortcomings^1^ and vast differences in study design and analyses across these studies aside, both of these previous EEG studies measured event-related potentials elicited by stimuli presented individually for several hundreds of milliseconds (2 s in Belanova et al. 2018; 600 ms in Faghel-Soubeyrand et al. 2024) in the context of *recognition* tasks. As such, both studies relied on data from low SNR trials (and vastly different amounts hereof^2^), and were thus neither designed nor appropriate to measure individual sub-processes separately.

Thus, currently, a systematic investigation into the different subprocesses involved in FIP *at the neural level* is missing entirely in the field of SR research. Behavioral experiments fundamentally lack the procedural controls to minimise the impact of any cognitive processes unfolding between stimulus onset and behavioral response measurement.^3^ Though fMRI measures of face processing are crucial to understanding the structures underpinning various FIP sub-processes, they lack the necessary temporal resolution to investigate distinct subprocesses in isolation.

### FPVS EEG as means to target FIP subprocesses

**Fig. 1:**
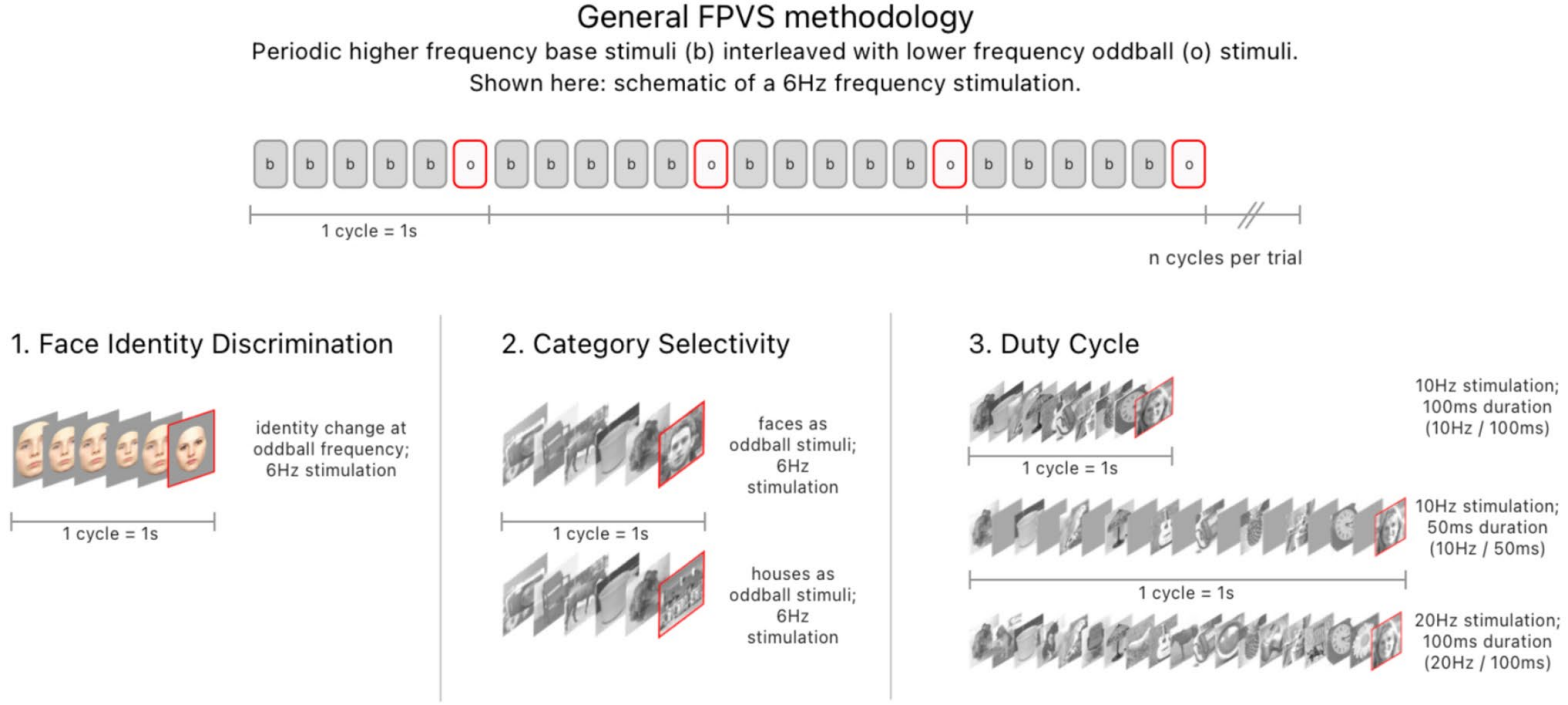
Fast periodic visual stimulation (FPVS): general methodology and experiments. Top: Schematic of a 6 Hz visual stimulation frequency involving presentation of five base stimuli (b) and one oddball stimulus (o) per cycle (second). Bottom: Schematic of the three experiments used in this study: 1 Face Identity Discrimination; 2. Category Selectivity; 3. Duty Cycle

Electrophysiological techniques, due to their higher temporal resolution (on the order of a few milliseconds, rather than seconds) are well-suited to target FIP subprocesses completed within less than a second. Fast Periodic Visual Stimulation (FPVS) coupled with EEG is an empirical method that involves measuring the unique neural response to ‘oddball’ stimuli embedded within a periodic train of other ‘base’ stimuli (for review, see Rossion et al. 2020). This paradigm capitalizes on variability between images, preserving only those neural responses that differ systematically between oddball and base stimuli. In the context of face processing for instance, FPVS EEG can thus provide relatively ‘pure’ (i.e., net of the varied image statistics) measures of FIP’s component sub-processes (Jacques et al. 2020; Rossion et al. 2018; Liu-Shuang et al. 2016; see Fig. 1).

First, face *detection* (involving simple visual awareness of the presence of a face stimulus within visual field) can be measured by demonstrating category selective neural responses to faces relative to other visual stimulus types (Liu et al. 2002; Rossion and Caharel 2011; Jacques et al. 2016). Experimentally, Jacques et al. (2016) employed FPVS EEG to show that the spatiotemporal distribution of neural responses generated by face oddballs are distinct from those generated by other object categories (e.g., house oddballs). Retter et al. (2018) used a similar paradigm to study face categorization with greater detail. They showed that it is fundamentally limited by faster presentation rates, rather than by shorter image durations. More specifically, neural oddball responses declined when the image presentation rate doubled, even though presentation durations were kept equal.

Second, the FPVS oddball paradigm can be used to measure the neural signature of identity *discrimination*. Sequential presentations of images of one repeated facial identity followed by one of a different facial identity (an identity ‘oddball’) generates neural responses at both the frequency of image presentation and—if the unique *identity* is discriminable from preceding images—the frequency of oddball presentation. These complementary neural responses have been used to provide empirical measures of face discrimination in prosopagnosia (e.g., Liu-Shuang et al. 2016) and to characterize individual differences among neurotypical observers (Stacchi et al. 2019a, b; Xu et al. 2017).

### Systematic neural investigation of FIP subprocesses in SRs

Departing from the aforementioned existing EEG studies (Belanova et al. 2018; Faghel-Soubeyrand et al. 2024), we adopted *FPVS EEG* to address the existing empirical void concerning the neural basis of SRs’ abilities. In doing so, we systematically targeted at the neural level two visuo-perceptual FIP subprocesses for which behavioral differences have been reported for SRs: face discrimination (e.g., Nador et al. 2021a, b) and face detection (Linka et al. 2022). Our methodological choice was motivated by FPVS EEG’s characteristic of being unbound by overt behavioral responses, and sufficiently sensitive, and hence efficient to measure multiple sub-processes within a short time (~ 40 min of data acquisition in total, over 3 experiments).

The present study served to answer distinct, but related questions. First, we sought to answer the question *Can we identify a neural correlate of SRs behaviorally reported FIP superiority?* Given the growing evidence that SRs’ enhanced perceptual processing affords more robust representations of facial identity, we sought to compare neural markers of their *identity discrimination* against neurotypical control observers’. To that end, in a first experiment, we used an identity oddball paradigm as an *implicit neural measure* of *identity discrimination* (Experiment 1) with which impaired FIP in acquired prosopagnosia, as well as behavioral ability and neural consistency have been measured previously (Liu-Shuang et al. 2016; Stacchi et al. 2019a, b; Xu et al. 2017). Next, in a pair of experiments conducted on the same samples of SRs and controls, we asked respectively: *Do SRs show differences in face vs. object categorization* (Experiment 2)*, and is their face detection affected differently by stimulus presentation parameters* (Experiment3)*?* Borrowing from previous reports in neurotypical and impaired observers, Experiment 2 investigates differences in category-selectivity (Jacques et al. 2016), while Experiment 3 determines the influence of changes in duration and rate of stimulus presentation on face categorization (Retter et al. 2018).

Through this combination of well-established FPVS paradigms measuring different aspects of visual perception implicitly within the same observers, we provide the most detailed description of F(I)P in SRs to date. What’s more, our findings are uncontaminated by attentional or decisional factors. To preview our findings, our study provides the first evidence to suggest that SRs’ abilities—at least at the group level—span multiple sub-processes, and do not arise fundamentally from advantages in *identity* processing. Rather, their advantages in later stages of FIP (e.g. identity discrimination or recognition) may be more likely carried forward from domain general advantages in perceptual processing.

## General methods

All procedures and protocols were approved by the Ethics Committee of the University of Fribourg (approval number 488) and conducted in accordance with the guidelines set forth in the Declaration of Helsinki. All volunteering participants provided informed consent and were not financially compensated for their participation.

### Participants

SRs reported in this study consisted of seven individuals (3 males, 4 females; see Table 1), with an average age of 39.3 years (*SD* = 9.5 years). These individuals met the criteria proposed previously by Ramon (2021) for lab-based SR identification: superior performance in at least two of three challenging tests of FIP probing face perception and face memory. More specifically, the tests used to determine SR status were the Facial Identity Card Sorting Test (FICST; Jenkins et al. 2011; Fysh et al. 2020; Stacchi et al. 2020), the Yearbook Test (YBT; Bruck et al. 1991; Stacchi et al. 2020), and the long version of the Cambridge Face Memory Test (CFMT + ; Russell et al. 2009). The group of control observers consisted of eight individuals (5 males, 3 females; 7 right-handed, one left-handed), with an average age of 37.5 (*SD* = 12.2), who were friends or colleagues of the experimenters, who had previously participated in various experiments (and were therefore not (potentially re)tested on the three FIP tests used as criteria) and self-reported normal abilities with no indication of superior processing abilities.

**Table 1:** Demographic information and behavioral test performance scores

ID	Handedness	Gender	Age	FICST	YBT	CFMT
MH1	R	F	41	0	19	73
MA1	L	F	26	3	16	88
DG1	R	M	45	0	18	82
CG1	R	F	24	0	16	85
MM1*	R	M	45	1	22	97
SS1*	R	M	50	0	20	101
KC1	R	F	44	1	17	91

Individual SRs’ scores on three tests of face identity processing used for lab-based SR identification (Ramon 2021). Asterisks indicate SRs originally reported by Ramon (2021). Indicated ages refer to participants’ age at the time of behavioral testing

### EEG recording

We recorded observers’ EEG data using a 64 Ag/AgCl active electrode (Biosemi Systems) extended 10–20 system, along with 4 additional external (ocular) electrodes, a common mode sense (CMS), and a driven right leg (DRL). Two of the external electrodes were placed, one each, roughly 1 cm from observers’ left and right temporal canthi, and horizontally aligned with their pupils while looking straight ahead. These recorded horizontal electro-oculograms (HEOG). The other two external electrodes were positioned over the right maxilla and frontal bone, in vertical alignment with the observer’s right pupil to measure vertical electro-oculograms (VEOG).

### Stimuli

Over the course of the three experiments, images were displayed on a portable EEG system, concurrently under development. Over that time, we recalibrated display software to run on 3 different 60 Hz LCD monitors (all equally gamma-corrected) to the first 4 observers we recorded, before settling on a Samsung SyncMaster 2233RZ-3D LCD monitor (Wang and Nikolic′ 2011) (average luminance ca. 110 cd/m^2^; 1680 × 1050 px resolution; 60 Hz refresh rate; NVIDIA® GeForce® GTX 1660 graphics card), against a neutral gray background. All stimuli were taken from the database available through the Rossion Face Categorization Lab (https://face-categorization-lab.webnode.page/people/bruno-rossion/), and were presented via a bespoke stimulus display software for EEG experimentation kindly provided by Bruno Rossion.

### Procedure

Before beginning the experiment, observers were instructed that they could begin each trial whenever they were ready, by pressing the spacebar; until then, only a blank grey screen would be displayed. Once the spacebar was pressed, a black fixation cross (30 pixels in diameter) would appear at the center of the screen for 2–5 s (randomly determined) signifying that stimulation with a sequence of images (and an orthogonal task) would begin shortly. The cross’ initial appearance also signified to observers that they were meant to begin minimizing their movements (e.g., blinking, swallowing) and remain as still as possible throughout the trial.

Once a trial began, the fixation cross would remain displayed throughout, but, at random intervals (though always ten times per trial, and never less than 2.5 s apart), it would become red for 500 ms. Observers’ only task throughout all trials in all experiments was to monitor the cross for such changes and press the spacebar as quickly as possible whenever one was noted. Critically, these changes were completely unrelated in time or content to the simultaneously presented image sequences, ensuring task orthogonality from base and oddball image displays. Following each image sequence, the fixation cross would remain for another random 2–5 s interval before disappearing, leaving only the blank gray screen. At this point, observers could prepare themselves for the next trial, before initiating it with the spacebar.

### Analyses

#### EEG preprocessing

EEG data were preprocessed using the Letswave 7 library (https://letswave.cn/) in a Matlab environment (2023b, Mathworks Inc.). To begin with, we interpolated any channels deemed noisy (by visual inspection) using their three nearest neighbors (affecting one channel each, for 3 participants), then down-sampled by a factor of four (from 1024 to 256 Hz) and notch-filtered the data at 50 Hz (and its first two Harmonics; 2 Hz filter width) to remove line noise. Afterwards, we applied a fourth-order Butterworth bandpass filter with high and low cutoffs of 100 and 0.05 Hz, respectively. Ocular artifacts were then removed using the four external electrodes according to Gratton et al. (1983) algorithm, and finally re-referenced to the mean across electrodes.

Next, we segmented the data into sections containing one trial's worth of data each. This involved trimming off the EEG data recorded before and after each trial's full stimulation sequence, as well as during the contrast ramp-up and -down at the beginning and end respectively (see the individual experiments’ Procedure section for the exact stimulation sequence timings used per experiment). We also computed single-epoch data, where each individual trial was further divided into sections containing the data recorded during one period (one second) of oddball stimulation.

##### Whole-trial data

For each observer, we took the Fourier transforms of every whole trial's EEG data, yielding the amplitude at each resulting frequency**.** Afterward, we averaged across trials for each condition of each experiment. Fourier transforms’ precision is limited to measuring frequencies at intervals corresponding to the number of time points divided by the sampling rate (256 Hz). Pre-processed trials in Experiments 1 and 2 were ~ 56 s long (14,337 samples, after removing contrast ramps and blank intervals), resulting in 0.0179 Hz intervals between measured frequency bins. Since Experiment 3 had ~ 90 s long trials (23,041 samples), bins were 0.0111 Hz apart.

The resulting amplitude at each frequency bin was then baseline-corrected by subtracting the mean of the 20 nearest bins (10 above/below), excluding the two adjacent (one above/below) bins (see Rossion et al. 2020 for further details). (Note that means were computed immediately after Fourier transformation—and before baseline correction—to reduce potential edge artifacts.) We then averaged the baseline-corrected amplitudes over parieto-occipital electrodes (as in Rossion et al. 2020; Retter et al. 2021) including PO7, PO8, P7, P8, P9 and P10 to compute a bilateral face-selective ROI.

Within this ROI, we calculated the total ‘base’ and ‘oddball’ responses separately at each electrode and then averaged them. First, for base responses, we summed the baseline-subtracted amplitudes of all frequency bins that were multiples of the base frequency, up to 48 Hz. Then, for the oddball response, we summed the baseline-subtracted amplitudes of all frequency bins that were multiples of the oddball frequency—excluding any bins summed in the base response calculation—up to 48 Hz. In all experiments, base harmonics were defined as integer multiples of the base rate up to 48 Hz (also including adjacent bins). Adjacent bins were included in these sums since, due to monitor recalibrations between participants, the exact stimulation frequency varied between participants by up to one bin width (0.0179 Hz), as described earlier. Oddballs occurred at 1 Hz, so oddball response amplitudes were taken as the sum of all integer frequencies (and the two immediately adjacent frequency bins—one above and one below) excluding the base stimulation rate and its harmonics.

##### Single-epoch data

Having subdivided each observer's trials into one-second epochs, we took the Fourier transforms of each one. The resulting bin widths were 1 Hz in all three experiments (since sampling rate and epoch length were the same throughout). We baseline-corrected the resulting amplitudes as above (see *Whole-trial Data*). These segments were neither averaged, nor were any ROIs derived for them.

#### Bayesian ANOVA

To quantify between-*Condition* and between-*Group* differences in the amplitude of base stimulation and oddball response, we ran two-way mixed Bayesian ANOVAs (JASP, Version 0.17.3, 2023) with *Group* as a between-subjects factor, and *Condition* as the within-subjects factor. In each experiment, this yielded two Bayesian ANOVAs: one considering the summed amplitudes for the base (presentation rate) harmonics as its dependent variable, and the other using the oddball harmonics as its dependent variable. In Experiment 1 (*Identity Discrimination*), this yielded two 4 (3 Hz, 6 Hz, 9 Hz, 12 Hz) X 2 (*control*, *SR*) ANOVA designs; in Experiment 2 (*Category Selectivity*), this yielded two 2 (*Faces*, *Houses*) X 2 (*control*, *SR*) ANOVA designs; finally, in Experiment 3 (*Duty Cycle*), this yielded two 3 (10 Hz 100%, 10 Hz 50%, 20 Hz 100%) X 2 (*control*, *SR*) designs.

#### Logistic classification

The Bayesian ANOVAs above only consider the sum of amplitudes across frequencies, thus overlooking more granular effects of group or condition at any particular frequency. Moreover, FIP could proceed along different neural pathways—and thus produce different scalp distributions—for SRs and controls, not accounted for in the preselected electrodes that make up the bilateral face-selective ROI under consideration (Nador and Ramon 2021a). Thus, we sought to employ an electrode agnostic method for assessing FIP with greater granularity: considering the influence of amplitudes at all scalp electrodes at each individual frequency (rather than only the sum across frequencies). So, in each experiment—and separately for each observer—we sought to determine whether the pattern of amplitudes in the Fourier spectrum across the 64 scalp electrodes predicts experimental conditions with better than chance accuracy. To that end, we trained logistic classifiers on random samples of 60% of each observer’s epoched data (see Single-Epoch Data, above) using threefold cross validation.

Model parameters were chosen by Ridge regularization, which penalizes the magnitude of the coefficients to prevent overfitting. The training data underwent scaling to unit variance and Principal Component Analysis (PCA) with retention of 20 components. For penalization, a value was selected using an internal threefold cross validation procedure (on the training data), considering 10 logarithmically spaced values ranging from 10^–3^ to 10^3^. The final (trained, cross-validated) models were tested on the remaining 40% of withheld data. In Experiments 1 and 3, involving more than two conditions, we trained one-versus-one logistic classifiers, which themselves inform a separate binary classifier for every possible pair of conditions. The final prediction is made by a majority voting scheme among all binary classifiers. In Experiment 2, with only two conditions, we trained a single one-versus-one logistic classifier. In all three experiments, though, the procedure was repeated 100 times with different random allocation of trials to the training and testing data. The final evaluation involved calculating the median accuracy across the 100 repetitions for each subject.

## Experiment 1: Face identity discrimination

As described above, the FPVS identity oddball paradigm effectively measures the brain’s ability to implicitly discriminate facial identities, by comparing neural responses to a novel identity embedded within periodic presentations of a repeated facial identity (Liu-Shuang et al. 2016; Stacchi et al. 2019a, b; Xu et al. 2017). In this experiment, we systematically varied face image presentation rate (i.e., from 3 to 12 Hz) to test whether neural responses to such ‘identity oddball’ stimuli would differ between Controls and SRs.

If, as a group, SRs were to show substantially different identity oddball neural response amplitudes from controls, this could provide a basis for their superior abilities, which generally includes face identity discrimination. In general, identity oddball responses should be(come) attenuated for conditions with stimulation frequency exceeding an individual’s FIP speed. So if, as a group, SRs have some advantage in FIP speed over controls, their oddball responses should persist in conditions with successively shorter presentation times (but also, quicker presentation rates).

### Method

#### Stimuli and procedures

This experiment made use of 50 images, taken of unique facial identities (half male and half female), all from a full-frontal viewpoint, under standardized conditions (cf. Fig. 1). They were each then digitally cropped to remove external features, rescaled so that all had equal heights, and shown against a neutral grey background. Rather than controlling for any other low-level stimulus features, we randomly varied them between images, thus ruling out any systematic contributions (for examples see Thorpe et al. 1996; Crouzet et al. 2010; Földiák et al. 2004; Rossion et al. 2015; Retter et al. 2020; Retter et al., 2021). For each stimulus presentation, image size, luminance, and contrast was modulated to increase variability along these dimensions (across presentations). Each image was assigned a display size between 80 and 120% (in 5% increments) of its original size, and a luminance value ± 10% (in 2.5% increments) of the original. Images’ contrasts varied sinusoidally from 0 to 100% over time upon each presentation.

Each trial comprised a 60 s stimulation sequence of face images, displayed at one of four presentation rates throughout: 3 Hz, 6 Hz, 9 Hz, or 12 Hz. The first image in each sequence was displayed at 0 luminance contrast; subsequent images’ contrasts increased linearly, reaching full contrast after 2 s, and decreasing back to 0 in the last 2 s. Every second of a sequence ended with an identity oddball, preceded by images of the same base face (together constituting one epoch). Four sequences (2 male, 2 female) of each type were shown to each observer in random order.

### Results

#### Bayesian ANOVAs

While the Bayesian ANOVA in *Base Response* amplitudes decisively prefers all alternative models in Table 2 to the null model (R^2^_posterior_ (13) = 0.717), considering each model separately yields decisive evidence in favour of modeling the data based only on the *Presentation Rate* main effect (Log(BF_M_ = 1.312). Moreover, the model including *Group* and *Presentation Rate* main effects together (Log(BF_M_) = 0.622) would be substantially supported as well. However, matched model comparisons (assessing each factor’s effect by comparing all models including it vs. excluding it) favour including *only* the main effect of *Presentation Rate* (Log(BF_Pres. Rate_) = 27.495). So, overall, there is, at best, limited support for a *Group* main effect, suggesting that the data are best accounted for by the main effect of *Presentation Rate* alone. Post hoc comparisons for the main effect of *Presentation Rate* reveal that 3 Hz base-rate images produced the highest amplitude *Base Response* harmonics, followed by 6 Hz and then 9 Hz, while 9 Hz and 12 Hz were effectively equivalent. Overall, this suggests that faster image presentation induces successively less prominent *Base Response* amplitudes at relevant harmonics, at least up to 9 Hz, beyond which neural responses seem to reach their floor.

**Table 2:** Model comparisons for summed base image and identity oddball harmonics

	P(M)	P(M|data)	Log(BF_M_)	Log(BF_10_)	error%
Base image response models					
Null model (subject + random slopes)	0.2	6.422 × 10^–13^	− 26.688	.000	
Pres. Rate	0.2	0.481	1.312	27.343	1.085
Pres. Rate + Group	0.2	0.318	0.622	26.927	1.172
Group + Pres. Rate + Group✻Pres. Rate	0.2	0.201	0.005	26.469	1.358
Group	0.2	2.732 × 10^–13^	− 27.542	− 0.855	0.728
Identity oddball response models					
Null model (subject + random slopes)	0.2	0.397	0.968	0	
Group	0.2	0.311	0.592	− 0.243	1.100
Pres. Rate	0.2	0.140	− 0.432	− 1.045	0.602
Group + Pres. Rate	0.2	0.112	− 0.682	− 1.263	1.481
Group + Pres. Rate + Group✻Pres. Rate	0.2	0.040	− 1.793	− 2.296	1.264

Exhaustive comparisons of possible models in the Face Identity Discrimination experiment, conducted separately for base image (top) and identity oddball (bottom) responses, containing every possible combination of factors (including main/interaction terms), against the null model including only subject and random slope terms for all repeated measures factors

Meanwhile, Bayesian ANOVA in *Oddball Responses* most strongly supports the null model (Log(BF_Null_) = 0.968, R^2^_posterior_ (13) = 0.298); See Table 2). Though there is limited support for modelling the data by a *Group* main effect (Log(BF_Group_) = 0.592), further matched-models analysis of separate effects actually favors *excluding* the main effect of *Group* (Log(BF_incl_) = − 0.552), as well as *Presentation Rate* (Log(BF_incl_) = − 1.292). Similarly, the matched-models analysis of separate effects decisively finds no *Group* by *Presentation Rate* interaction. Overall, these results most strongly support the null hypothesis: there is no main effect of *Presentation Rate* or *Group* on *Oddball Response* amplitude, and the effect of *Presentation Rate* on *Oddball Response* does not depend on *Group* (i.e., the two do not interact).

#### Logistic classification

**Fig. 2:**
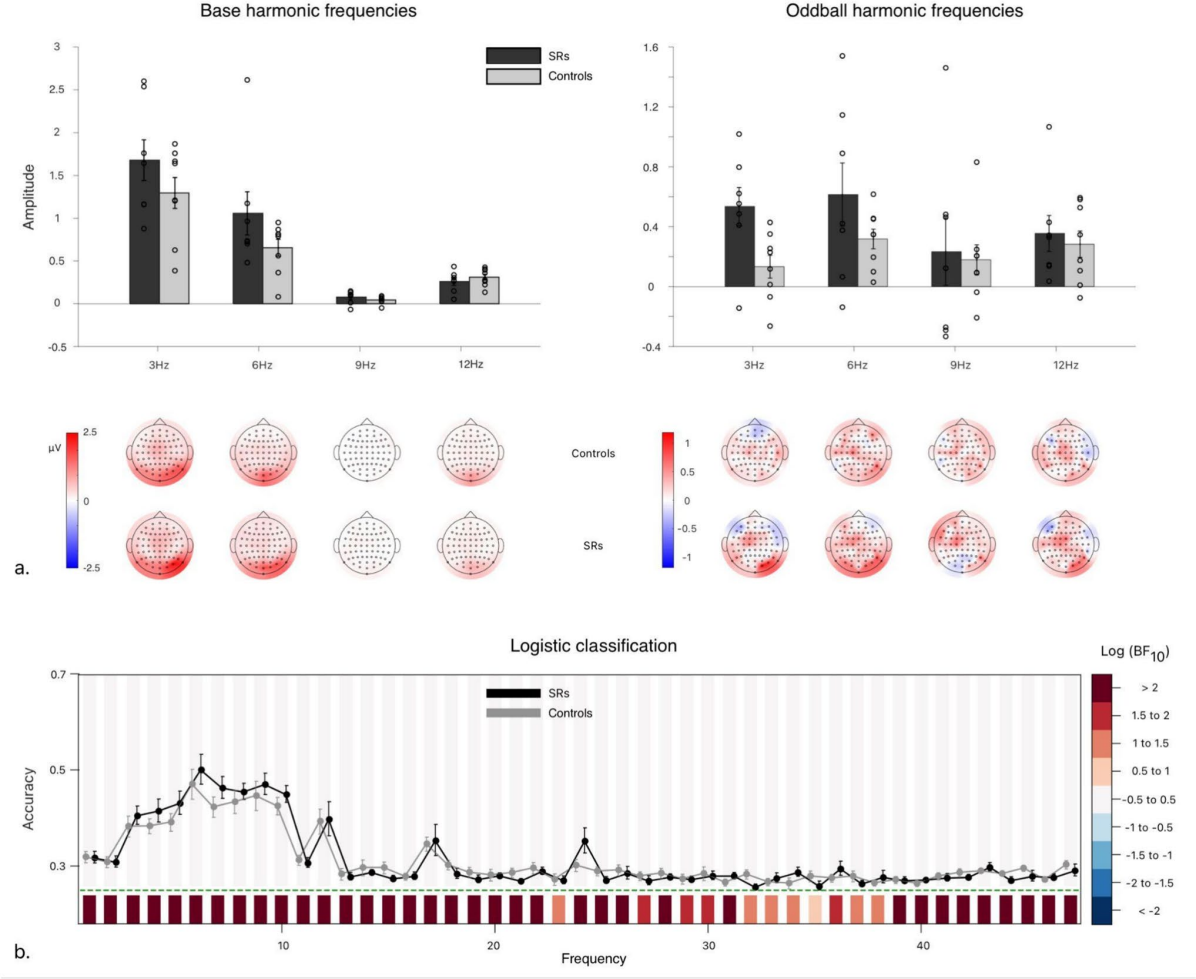
Experiment 1: Face Identity Discrimination. **a** First row: Bar charts of group-means of the sum of frequency-baselined amplitudes of the base (left) and oddball (right) harmonic frequencies taken over occipitoparietal ROI (Bayesian ANOVAs, in general methods). Error bars correspond to ± 1 SEM. Second row: Topographies of controls’ (top) and SRs’ (bottom) group-averaged, frequency-baselined amplitudes (in µV) for base (left) and oddball (right) harmonic frequencies, interpolated over scalp electrodes. Note: axes are not equivalent for base and oddball frequencies; these should not be compared directly, as different numbers of frequency bins are summed to determine each total amplitude. **b** Plot of mean logistic classification accuracy, repeated at each frequency from 1 to 47 Hz, using scalp electrodes as potential ‘features’ for the machine learning algorithm. Error bars about grand average correspond to standard errors of the mean. Shaded regions above chance accuracy (green dotted lines) correspond to log Bayes Factors for Group comparisons; regions below correspond to log Bayes Factors comparing the Grand average against chance

Considering the group-averaged logistic classification of 1 s epochs’ conditions (conducted separately for each observer), we find that observers’ neural responses to oddball stimuli were predicted with greater than chance accuracy over harmonics up to 13 Hz (Fig. 2b). However, no significant between-group differences in classification accuracy were apparent. Notably, there was no specific difference between any two conditions, as classification accuracies remained comparable regardless of which condition was treated as signal, even at the largest value of Cohen’s d between groups.

### Discussion

SRs’ neural responses to identity oddballs were effectively no different from controls’, nor did they depend on *Presentation Rate*. This result, and those of other recent ERP studies (Belanova et al. 2018; Faghel-Soubeyrand et al. 2024) suggest that SRs’ FIP advantages do not arise from facial *identity* discrimination specifically. Rather, our results align with a predominantly perceptual locus for SRs’ advantages in processing both face and non-face stimuli. SRs could simply represent an extreme case of the broader brain-behavior relationship, in which early ERPs vary as a function of behavioral performance on standardized testing of face memory (Schroeger et al. 2023).

Moreover, these results support—even if only weakly—the possibility that SRs’ processing advantages arise from facilitated face *image* (as opposed to identity) processing. This experiment provides limited evidence that SRs’ neural responses to face images—though not identity changes—were greater than controls’. However, group differences obscured by the limited range of stimulus types in this experiment could potentially be revealed by comparing neural responses to more naturalistic face and non-face images.

As discussed earlier, processing a facial identity requires that multiple prior subprocesses successfully unfold first; for instance, faces cannot be identified before they are perceived, and properly categorized as faces. Indeed, 9–12 Hz presentation rates—where base responses reach their floor—may encroach on early visuocortical ERP latencies like the C1 and P1 components. Consequently, a proper evaluation of SRs’ abilities should further analyze subprocesses beyond those that are strictly identity-bound, and occur earlier in visual processing.

Finally, since logistic classification found similar accuracy for SRs and controls, it seems unlikely that there is any substantial difference between the *kinds* of processing in which both groups engaged at any specific harmonic frequency. So, whatever SRs’ advantage in FIP, it would seem to stem from the same subprocesses that neurotypical control observers possess.

## Experiment 2: Category selectivity

The results of Experiment 1 suggest that differences in facial identity discrimination—at least at the group level—do not seem to explain FIP differences between SRs and controls. As discussed from the outset, SRs’ generalized advantages could stem from facilitation of ‘earlier’ subprocesses (e.g., face categorization) preceding identity discrimination. Since FPVS can isolate categorization from other early subprocesses (c.f. Rossion et al. 2020), we adopted it to compare face categorization directly between the same samples of Controls and SRs reported in Experiment 1.

To test whether our SRs’ superior abilities are selective for faces (relative to houses) during categorization, we compared neural responses of SRs to naturalistic face versus house oddball images embedded in periodic presentations of images of various object categories (Jacques et al. 2016). In addition to manipulating oddball types (face vs house), we also varied the contrast of stimuli in the house oddball condition to maximize the oddball manipulation’s ability to generate between-Group differences. If SRs were to show enhanced face oddball responses relative to controls, this would suggest that their processing advantage is already measurable during face categorization as the entry point of FIP. Meanwhile, if SRs differed from controls in terms of their response to base stimuli, this would further support the hypothesis that, whatever their processing advantage over controls, it is not specific to faces.

### Method

#### Stimuli and procedures

Observers viewed naturalistic grayscale images of faces (counterbalanced to include 25 males and 25 females), houses, cats, dogs, horses, birds, flowers, fruits, vegetables, houseplants, phones, chairs, cameras, dishes, guitars and lamps (obtained from http://face-categorization-lab.webnode.com/resources/natural-face-stimuli/) against a neutral grey background (50% maximum pixel luminance). The 200 × 200 pixel images were scaled up to 280 pixels and presented at approximately 70 cm viewing distance, thus subtending ~ 5° visual angle.

To preserve only natural variation amongst face images, we did not implement any luminance normalization within or between image categories in the Face condition. However, we modulated image contrast randomly between images in the House condition, such that each was displayed at 80–100% of full contrast. This same manipulation was *not* applied to the Face condition, consequently maximizing the experiment’s ability to generate larger oddballs to faces than to houses, but still *equally greater in both groups*. Faces had a mean RMS contrast of 0.247 ± 0.032 (mean ± 1 SD); houses’ RMS contrast (in Experiment 1) was 0.239 ± 0.035 and objects’ mean RMS contrast was 0.238 ± 0.041.

In this experiment, observers were shown eight trials consisting of 60 s-long sequences of images, displayed at 6 Hz. Each trial began and ended with an effective luminance contrast of 0, linearly ramping from 0 to full (over the first 5 s of each trial) and back to 0 (over the last 5 s). Every second of each trial, five “base” images of non-house objects, followed by an “oddball” image (either a face or a house), would be displayed periodically, forming two conditions based on the oddball’s category. The two types of oddball trials were blocked (i.e. one oddball condition per block) such that 4 of each were shown to every observer, in a random order within each block, always beginning with the face block.

Throughout each image sequence, and for 2–5 s before and afterwards (randomly), a black 1.2° central fixation cross was displayed, overlaid on the images. The cross would periodically turn red for 1 s at random intervals (but always 10 times per sequence); observers were instructed to press the spacebar as quickly as possible every time it turned red—preferably before it returned to black.

### Results

#### Bayesian ANOVAs

Bayesian ANOVA in *Base Response* reveals that the most parsimonious explanatory model includes only the effect of *Group*, though it provides no more parsimonious explanation of the data than the null model (See Table 3). Overall, the ANOVA accounts for 72% of the variance in *Base Response* (R^2^_Posterior_ (13) = 0.724). Matched-models analyses decisively favour excluding the main effect of *Stimulus Type* (Log(BF_Incl_) = − 0.982), through there is limited evidence for including or excluding of the main effect of *Group* (Log(BF_Incl_) = 0.082), or its interaction with *Stimulus Type* (Log(BF_Incl_) = − 0.365). Overall, the effect of *Group* provides no more parsimonious explanation of the data than the Null model, and provides no advantage in explanatory power relative to matched models.

**Table 3:** Model comparisons for summed base image and category oddball harmonics

	P(M)	P(M|data)	Log(BF_M_)	Log(BF_10_)	error %
Base image response models					
Null model (subject + random slopes)	0.2	0.319	0.628	0.000	
Group	0.2	0.342	0.733	0.070	2.771
Stim. Type	0.2	0.131	− 0.506	− 0.890	7.020
Stim. Type + Group	0.2	0.117	− 0.637	− 1.005	7.708
Stim. Type + Group + Stim. Type ✻ Group	0.2	0.091	− 0.916	− 1.225	1.832
Category oddball response models					
Null model (subject + random slopes)	0.2	2.142 × 10^–4^	− 7.063	0.000	
Stim. Type	0.2	0.579	1.705	7.903	1.029
Stim. Type + Group	0.2	0.269	0.387	7.137	1.273
Stim. Type + Group + Stim. Type ✻ Group	0.2	0.152	− 0.335	6.564	5.193
Group	0.2	9.274 × 10^–5^	− 7.899	− 0.836	1.497

Exhaustive comparisons of possible models in the Category Selectivity experiment, conducted separately for base image (top) and category oddball (bottom) responses, containing every possible combination of factors (including main/interaction terms), against the null model including only subject and random slope terms for all repeated measures factors

Meanwhile, Bayesian ANOVA revealed that the variance in *Oddball Response* is best explained by the model including only the main effect of *Stimulus Type* (See Table 3). Though all alternative models are decisively preferable to the Null, matched-model comparisons decisively favor including only the effect of *Stimulus Type* (Log(BF_Incl_) = 7.925), while strongly opposing inclusion of either its interaction with *Group* Log(BF_Incl_ = − 0.573), or the main effect of *Group* (Log(BF_Group_) = − 0.766). Overall, the model-averaged R^2^ accounts for 54% of the variance in *Oddball Response* amplitudes (R^2^_Posterior_ (13) = 0.540), and these results clearly suggest that the main effect of *Stimulus Type* is the only one of any explanatory importance.

#### Logistic classification

**Fig. 3:**
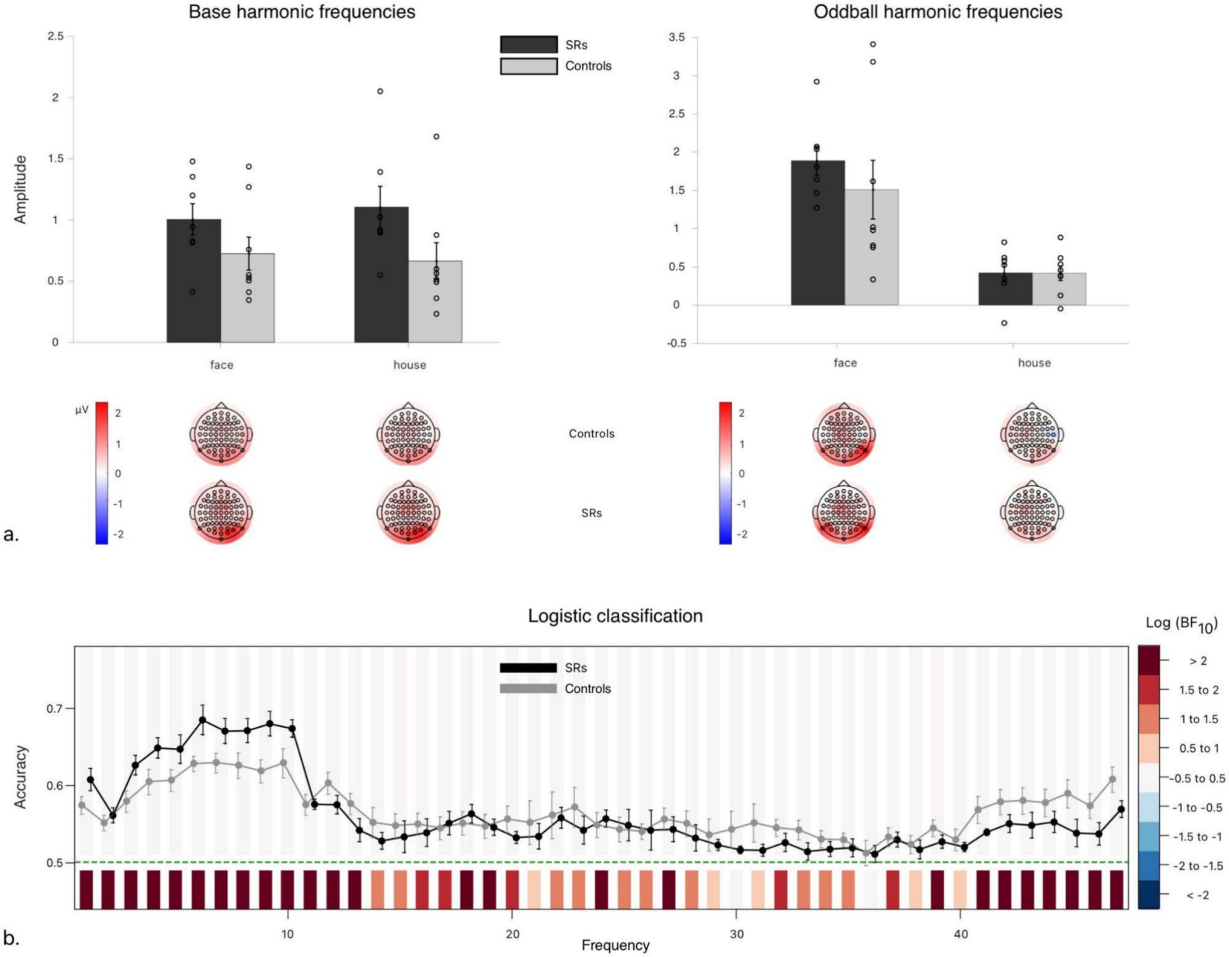
Experiment 2: Category Selectivity. **a** First row: Bar charts of group-means of the sum of frequency-baselined amplitudes of the base (left) and oddball (right) harmonic frequencies taken over occipitoparietal ROI (Bayesian ANOVAs, in general methods). Error bars correspond to ± 1 SEM. Second row: Topographies of controls’ (top) and SRs’ (bottom) group-averaged, frequency-baselined amplitudes (in µV) for base (left) and oddball (right) harmonic frequencies, interpolated over scalp electrodes. Note: axes are not equivalent for base and oddball frequencies; these should not be compared directly, as different numbers of frequency bins are summed to determine each total amplitude. **b** Plot of mean logistic classification accuracy, repeated at each frequency from 1 to 47 Hz, using scalp electrodes as potential ‘features’ for the machine learning algorithm. Error bars about grand average correspond to standard errors of the mean. Shaded regions above chance accuracy (green dotted lines) correspond to log Bayes Factors for Group comparisons; regions below correspond to log Bayes Factors comparing the Grand average against chance

Though observationally, it might appear that compared to controls, SRs’ neural data better classify stimulus conditions (Fig. 3b), there is no statistically significant difference in accuracy between groups. So, whatever the basis for SRs’ advantage in stimulus processing (evidenced by the main effect of Group in the Bayesian ANOVA in base harmonic responses), it does not appear to be face-specific (Fig. 3a). Even though faces produce a clearly unique oddball signature (as evidenced by the main effect of *Condition* in the Bayesian ANOVA in *Oddball Response*), this subprocess seems to unfold comparably for SRs and control observers.

### Discussion

This experiment’s results overarchingly suggest that face oddballs are categorized distinctly from houses among numerous other object categories, but no more so by SRs than Controls. This is supported both by greater accuracy for faces than houses in classification, and by the main effect of *Condition* revealed by ANOVA in *Oddball Response* amplitudes at relevant harmonic frequencies.

More broadly, these findings imply that any behavioral advantage in FIP that SRs might have over neurotypical individuals is unlikely to arise consistently from face categorization specifically. In agreement with previous research (e.g., Faghel-Soubeyrand et al. 2024), and Experiment 1, SRs’ FIP advantages seem to stem from a broader or more general perceptual advantage preceding face categorization.

## Experiment 3: Duty cycle

Having examined the ability of SRs and controls to implicitly and potentially differentially categorize face images, we next sought to examine the temporal dynamics of this subprocess. More precisely, our results thus far could be explained by SRs exhibiting a broader perceptual advantage in processing *speed* over controls. This in turn could account for their general advantage in categorization we found in Experiment 2, as well the absence of a *Group* main or *Group* by *Base-Rate* interaction effects on base responses found in Experiment 1. Therefore, in Experiment 3, we sought to directly test whether presentation parameters related to processing speed (i.e., image duration, presentation frequency, or both) might contribute to this advantage. To that end, we adapted the FPVS paradigm used for image classification (Jacques et al. 2016) to measure the effects of halving image duration, while maintaining the same presentation frequency, relative to doubling the presentation frequency—which also halves duration (Retter et al. 2018).

### Method

#### Stimuli and procedures

Using the same stimuli as the previous (Category Selectivity) experiment’s Face condition, and the same fixation cross task, observers viewed four 90 s sequences of face oddball images (1 Hz) among non-face objects in each of 3 conditions (12 trials total). Each full trial sequence began with a 3 s linear luminance contrast ramp from 0 to full and ended with a similar ramp from full to 0.

The conditions were each defined by a unique combination of presentation rate and stimulus duration: in the first, images were presented at 10 Hz, for 100 ms (i.e. with a 100% duty cycle); in the second, images were still displayed at 10 Hz, but their durations were halved (50 ms each, for 50% duty cycle) leaving a blank grey screen between image presentations; in the third condition, images were presented at 20 Hz, (i.e., 50 ms duration, 100% duty cycle). Regardless of condition, every second within each sequence terminated with a face stimulus, thus a category oddball.

### Results

#### ANOVAs

The Bayesian ANOVA in *Base Response* amplitudes most decisively favors the model including only main effects of *Group* and *Duty Cycle* over the Null model (see Table 4). However, all alternative models were decisively preferred over the null. Examination using matched models analysis decisively favors including the main effect of *Duty Cycle* (Log(BF_Condition_ = 10.496). There was no clear evidence regarding the main effect of *Group* (Log(BF_Group_ = 0.290), but enough to strongly exclude its interaction with *Duty Cycle* (Log(BF_Condition_) = − 0.641). Post hoc comparisons of *Duty Cycle* conditions revealed that halving image duration decisively reduced *Base Response* amplitudes, and doubling presentation frequency further substantially reduced them. Overall, while the most parsimonious overall model includes all main effects, the only one of any real consequence seems to be that of *Duty Cycle* (and not *Group*).

**Table 4:** Model comparisons for summed base image and face oddball harmonics

	P(M)	P(M|data)	Log(BF_M_)	Log(BF_10_)	error %
Base image response models					
Null model (subject + random slopes)	0.2	1.055 × 10^–5^	− 10.073	0.000	
Duty Cycle + Group	0.2	0.439	1.143	10.637	1.609
Duty Cycle	0.2	0.329	0.673	10.347	0.634
Duty Cycle + Group + Duty Cycle ✻ Group	0.2	0.232	0.187	9.996	1.648
Group	0.2	1.068 × 10^–5^	− 10.061	0.012	0.560
Face oddball response models					
Null model (subject + random slopes)	0.2	1.455 × 10^–6^	− 12.054	0.000	
Condition	0.2	0.495	1.368	12.738	0.743
Condition + Group	0.2	0.395	.962	12.513	1.853
Condition + Group + Condition ✻ Group	0.2	0.109	− 0.714	11.225	4.306
Group	0.2	5.946 × 10^–5^	− 12.485	− 0.411	0.587

Exhaustive comparisons of possible models in the Duty Cycle experiment, conducted separately for base image (top) and face oddball (bottom) responses, containing every possible combination of factors (including main/interaction terms), against the null model including only subject and random slope terms for all repeated measures factors

The Bayesian ANOVA in *Oddball Response* amplitudes (see Table 4) reveals that the only alternative model not decisively preferable to the Null preserved the main effect of *Group* alone. Overall, matched model comparisons decisively favour including only the effect of *Duty Cycle* (Log(BF_Duty Cycle_ = 12.824), while strongly discounting its interaction with *Group* (Log(BF_Group_ = − 1.288). Meanwhile, there was inconclusive evidence concerning the main effect of *Group* (Log(BF_Group_ = − 0.225). Post hoc comparisons of conditions (collapsed across *Group*) decisively showed that only doubling the frequency reduced *Oddball Response* amplitude, whereas there was no evidence for any effect of simply halving image duration.

#### Logistic classification

**Fig. 4:**
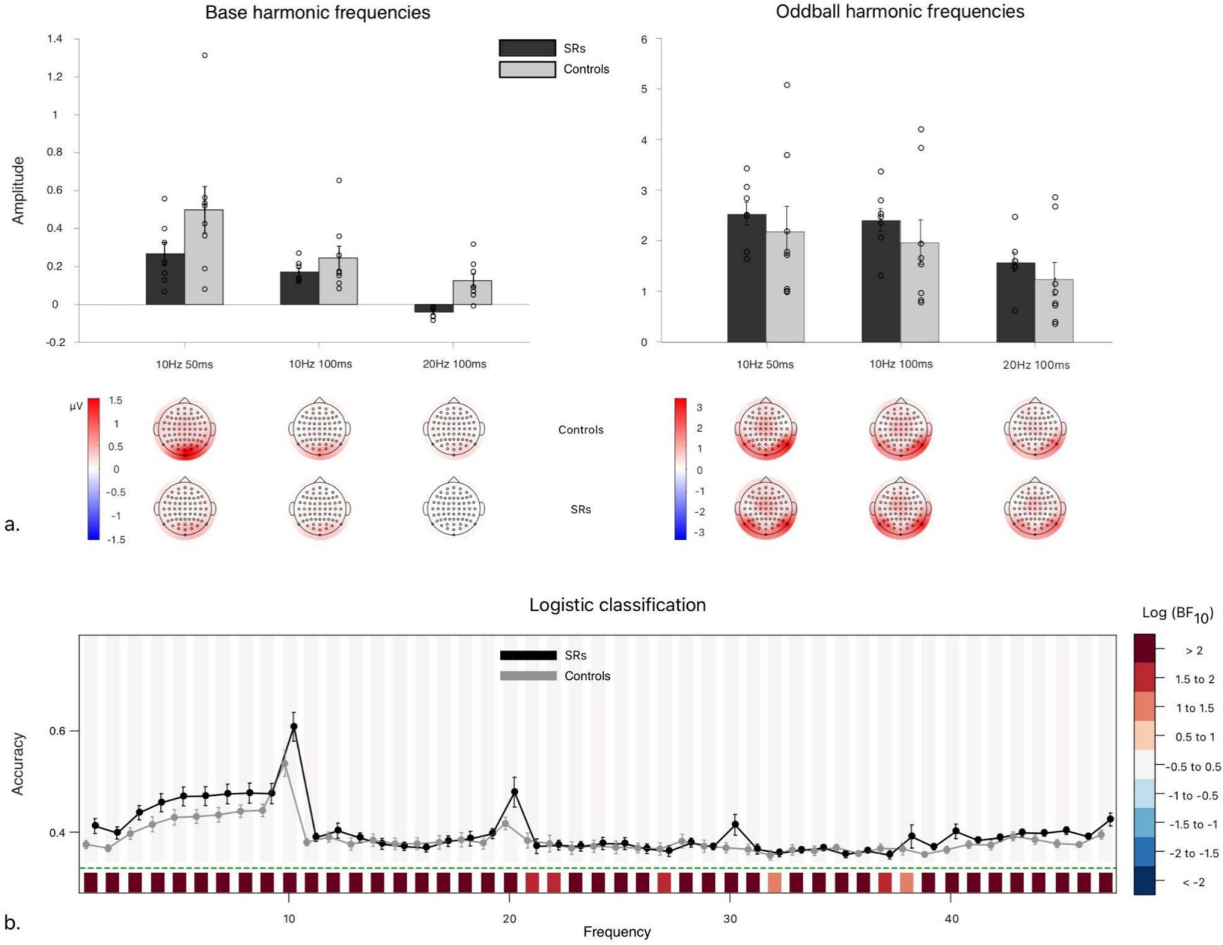
Experiment 3: Duty Cycle. **a** First row: Bar charts of group-means of the sum of frequency-baselined amplitudes of the base (left) and oddball (right) harmonic frequencies taken over occipitoparietal ROI (Bayesian ANOVAs, in general methods). Error bars correspond to ± 1 SEM. Second row: Topographies of controls’ (top) and SRs’ (bottom) group-averaged, frequency-baselined amplitudes (in µV) for base (left) and oddball (right) harmonic frequencies, interpolated over scalp electrodes. Note: axes are not equivalent for base and oddball frequencies; these should not be compared directly, as different numbers of frequency bins are summed to determine each total amplitude. **b** Plot of mean logistic classification accuracy, repeated at each frequency from 1 to 47 Hz, using scalp electrodes as potential ‘features’ for the machine learning algorithm. Error bars about grand average correspond to standard errors of the mean. Shaded regions above chance accuracy (green dotted lines) correspond to log Bayes Factors for Group comparisons; regions below correspond to log Bayes Factors comparing the Grand average against chance

Here we asked whether there is some group difference in processing speed of the face oddballs at any particular harmonic frequencies that can be predicted from the pattern of activity across all 64 scalp electrodes. Recall that the effect of halving image duration alone is visible by comparison of 10 Hz 100% and 10 Hz 50% conditions, while the effect of halving duration *and* doubling the frequency is measurable by comparison of the 10 Hz 100% and 20 Hz 100% conditions (see Fig. 4). Thus, if there were no difference between halving duration alone, and halving duration while also doubling frequency, then the resultant classification rates would be equal. To the extent that 10 Hz 100% epochs are more discriminable from 20 Hz 100% than they are from 10 Hz 50%, there is evidence that the ‘extra images’ present in the 20 Hz 100% epochs influence image processing irrespective of image duration. (It is instructive to think of each period of the 20 Hz 100% condition as equivalent to the 10 Hz 50% condition, but wherein the remaining 50% blank screen time is instead filled with an extra image).

The resultant classification accuracy extended over the same range of harmonic frequencies across both groups of observers (Fig. 4b). Moreover, halving duration alone generally induced fewer confusions than halving duration and doubling frequency at once. While, at the group level, the pattern of means alone could be taken to suggest that these confusions were less common among SRs, this was *not* statistically significant, even when examining the largest difference in classification accuracies between groups.

### Discussion

The results of the current experiment suggest that there is a robust difference between simply reducing image duration and increasing the delay between image presentations. The lack of any main effect of *Group* or interaction with *Condition* suggests that SRs and controls achieve image category selection via similar neural mechanisms and evince similar cross-condition changes in neural response to doubled image frequency and halved image duration.

## General discussion

Over the course of three experiments, we exploited the FPVS paradigm to isolate and assess FIP subprocesses with the potential to explain SRs’ abilities by differentiating the temporal profile of their neural responses to identity and category oddballs from controls’. Importantly, the specific protocols we employed are borrowed from previous studies examining FIP in the general population (Jacques et al. 2020; Rossion et al. 2018; Liu-Shuang et al. 2016), and in the past have only analyzed prespecified frequencies and electrodes (i.e., using ROI-based analyses). However, to account for the possibility that SRs could differ from controls at electrodes or frequencies not typically sensitive to FIP-related stimulation during FPVS, we also analyzed the full data set using a logistic classification machine learning algorithm.

### ANOVAs reveal perceptual, not FIP, advantages among SRs

We began by considering only the ROIs and frequencies standard to the FPVS paradigms employed here. In this limited view, whatever behavioral advantages SRs hold over controls, these do not arise from FIP per se. Rather, the scant evidence favoring group-level distinctions points to broader perceptual advantages.

In Experiment 1 *(Face Identity Discrimination*), Bayesian ANOVA in *Oddball Response* favored excluding *Group* as a factor. Where group differences did arise (bearing in mind there is limited evidence for this), they instead related to broader perceptual processing (Fig. 2). More concretely, neural identity-oddball responses did not distinguish SRs from controls, whereas neural responses to the image display-rate did, somewhat. On its own, however, this result does not speak to the face-specificity of the difference between SRs and controls, as both base and oddball images were faces. That is, display rate-dependent responses to faces could equally arise in response to other, non-face images.

With this in mind, Experiment 2 (*Category Selectivity*) explicitly compared face and house *Oddball Response* (among images taken from multiple other non-face categories) to determine whether their category-specificity could distinguish SRs from controls. But, while *Oddball Response* differed decisively between faces and houses (irrespective of *Group*), they were decisively similar between controls and SRs (Fig. 3 and Table 3).

Finally, in Experiment 3 (*Duty Cycle*) we sought to determine whether this potential difference in image processing was either rate- or duration-dependent, by either doubling the frequency or halving the presentation rate. In line with previous research (Retter et al. 2018), we find that while both rate and duration impact *Base Response* harmonic amplitudes, image availability is not a limiting factor in face oddball processing. Only doubling the frequency of base image presentation reduces the neural *Oddball Response*—even when controlling for presentation duration. Overall, this implies that the change in *Oddball Response* is more likely due to some combination of forward and backward masking of oddballs by temporally adjacent images (Retter et al. 2018). Moreover, there was strong evidence against any *Group* by *Duty Cycle* interaction, suggesting that—whatever this visual masking effect—it is most likely similar between SRs and controls.

Across experiments, these results can be summarized fairly succinctly: Whatever its neurological basis, *“super-recognition” most likely results from perceptual advantages, beyond the FIP subprocesses examined here*. Furthermore, this seems to hold true whether considering only occipitoparietal electrodes and harmonic frequencies analysed previously, or when including as many electrodes and harmonics as possible.

### Logistic classification reveals high degree of inter-individual variability

Although the ANOVA results discussed above suggest that the locus of between-group differences is perceptual, those analyses were based only on the limited set of electrodes making up the parieto-occipital ROI employed in previous research. So, the possibility remains that SRs’ FIP could still differ from controls at other loci. If these occurred at electrodes or frequencies not germane to neurotypical observers, it could be that while SRs do indeed differ from them, tests of conventional FIP neuromarkers are simply too narrowly focused to detect such differences. Therefore, we reanalyzed our data holistically—considering all available frequencies and electrodes—with a logistic classification machine learning algorithm.

Following this analysis, across the three experiments, the amplitude distributions across electrodes decisively distinguished between stimulus display conditions at *Base* and *Oddball Response* harmonic frequencies (see Figs. 2, 3, 4). However, we observed no between-group differences at any such harmonics in any experiment. So, whatever the *Group*-level amplitude distributions over the parieto-occipital ROI considered for Bayesian ANOVAs, they likely captured most sources of between-*Group* variability. Whichever other electrodes might have contributed to individual observers’ base and oddball responses, these varied enough between observers to act essentially as noise during classification. Nevertheless, this highlights a critical point corroborated in previous research: there is substantial inter- and intra-individual variability in FIP (Bobak et al. 2023; Stacchi et al. 2020; Fysh et al. 2020)—not only in the general population, but also amongst SRs (Nador and Ramon 2021a, b).

**Fig. 5:**
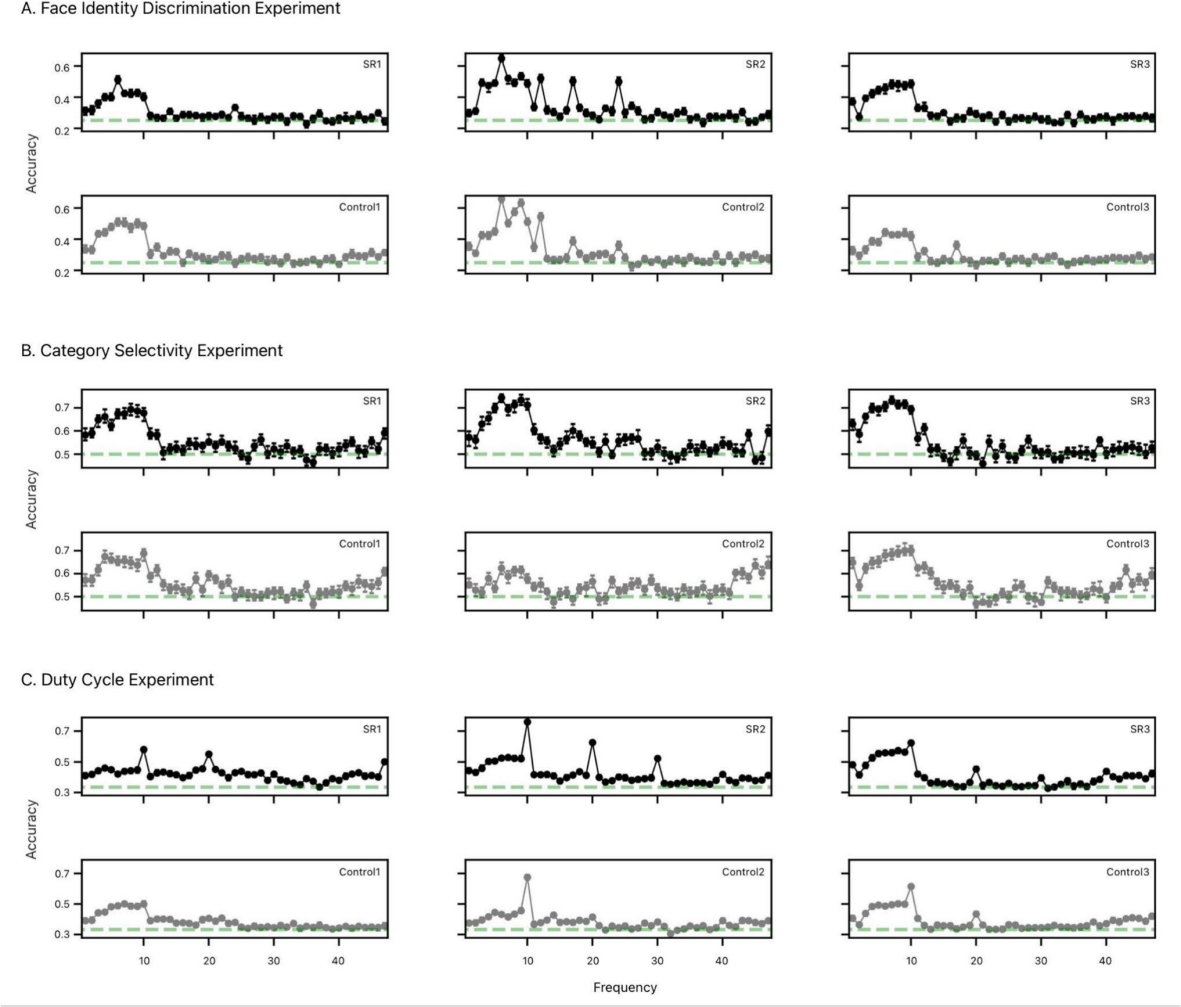
Individual differences in neural classification. **A** Logistic classification of 1 s epochs for a selection of 3 SR (top) and 3 control observers (bottom) in the Face Identity Discrimination experiment. The minimum, median and maximum classification accuracy are displayed, left to right. **B** Logistic classification of 1 s epochs for 3 SR (top) and 3 control observers (bottom) in the Category Selectivity experiment. The observers with the minimum, median and maximum classification accuracies in each group are displayed, left to right. **C** Logistic classification of 1 s epochs for 3 SR (top) and 3 control observers (bottom) in the Duty Cycle experiment

Breaking this result down by experiment, we see consistently that *Base Response* harmonic amplitudes classify observers at better than chance accuracy, but to varying degrees. To begin with, in the *Face Identity Discrimination* experiment (Experiment 2), there is wide variation in neural response classification between controls at both the base and oddball harmonic frequencies, as can be seen when comparing the highest, median, and lowest classification accuracies amongst controls, and also among SRs (Fig. 5). The variation between observers in each group implies that neural processes responsible for discriminating oddball from base facial identities (Experiment 1) do not induce consistent neural response amplitudes in all observers. Rather, there is enough variability between observers that it obscures whatever evidence might exist in support of either the null or alternative hypothesis.

Similarly, classification of neural responses in the *Category Selectivity* experiment (Experiment 2) decisively discriminates face from house oddballs in both controls and SRs, but not differentially as a function of group. So, while SRs’ neural responses to face versus house oddballs are decisively classified at better than chance accuracy, so too were controls’. As before, the extent of variability between observers was large enough to obscure the decision between null and alternative hypotheses. And again, the discrepancy between individual observers’ classification accuracies across frequencies bears this out (Fig. 5). Finally, classification of neural activity by stimulus presentation condition in the *Duty Cycle* experiment (Experiment 3) reveals quite similar patterns of accuracy—at both base and oddball harmonics—between groups (Fig. 4). Here again, we see that classification varies widely between both controls and SRs.

Overall, the classification results suggest that whatever neural processes distinguish a given SR from control observers, these are not common to all, or even most, SRs. Rather, it seems more likely that the *superior behavioral FIP abilities they display stem from individual-specific processing advantages*. So, whereas identity oddball responses may most accurately classify some SRs, others might be better classified by their ability to distinguish faces from other stimulus categories, and others still by their ability to do so extremely rapidly.

In sum, whatever the subprocess(es) responsible for between-group differences in neural responses, they cannot be isolated to a single harmonic frequency, or FIP subprocess. Per experiment, and across observers, oddball harmonic frequencies discriminated between stimulus display conditions. However, they were at best inconsistent in discriminating SRs from controls. Importantly, this was true both when considering the parieto-occipital ROI that we and previous researchers (see Rossion et al. 2020 for review) have employed, as well as when examining the pattern of activity across all electrodes, and at individual harmonic frequencies. This implies that SRs were probably not utilizing any process beyond those employed by controls during FIP. Otherwise, we would expect differences at some electrodes beyond those in the parieto-occipital ROI, that would have driven between-group differences in logistic machine learning classification at oddball harmonic frequencies. Instead, inclusion of the whole scalp topography revealed primarily that there is a substantial degree of variation between observers.

### Behavioral explanation for Super-Recognition

Considering the totality of our results, *no single electrophysiological FIP correlate distinguished SRs from controls*. However, general perceptual processing advantages aside, we cannot exclude task-related processing advantages as an explanation. In fact, several convergent lines of research suggest that SRs’ advantages are apt to be rather task-specific. Some have proposed that bespoke testing of a given task better predicts performance in real-world settings than aggregated testing across multiple abilities (e.g. Bobak et al. 2016, 2023; Nador et al. 2022; Ramon et al 2019a, b; Ramon 2021).

In the same vein, the present experiments are explicitly designed to isolate FIP subprocesses. So, in removing task-specific advantages, they inherently overlook any such basis for SRs’ superiority. For example, the tasks commonly used for lab-based SR diagnosis (Ramon 2021) require not only face processing ability, but also short term memory (e.g., to retain experimentally learned target identities in the CFMT +). Even tasks of simultaneous perceptual matching involve (to a degree) working memory (e.g. tracking sorted card groups in the FICST), and short-term memory (e.g. monitoring which pairs of faces have already been matched in the YBT). So, while the present experiments yield limited evidence of group-level differences, factors beyond visual FIP could still distinguish SRs from the general population, potentially in terms of behavioral task performance.

In this case, greater test sensitivity and the ability to identify SR sub-classes would provide a more complete, and more nuanced picture. To that end, the recently developed Berlin Test for Super-Recognizer Identification (beSure®; Ramon and Rjosk 2022) measures FIP using authentic forensic material, across various tasks and increasingly difficult real-life scenarios, with sufficient trial numbers to allow for descriptions of *various ability profiles among SRs*. For example, picking one facial identity out of a crowd from a short video would be facilitated not only by enhanced face processing abilities, but also from superior scanning, or visual search. And indeed, recent research supports the notion that even those individuals who excel at some tasks might still show enough intra-individual variation in ability across tasks to perform near the level of the population mean on others (Bobak et al. 2023; Stacchi et al. 2020; Fysh et al. 2020; Ramon and Vowels, 2025).

Arguably, then, group differences might be more readily uncovered using experimental paradigms that test task performance directly. Whereas here we employed FPVS experiments comprising an orthogonal task, future research might consider prompting responses to FPVS oddball stimuli directly and comparing against the same stimuli without task-dependence. This could be accomplished by replacing the orthogonal task employed here with oddball stimulus-related judgments. If indeed SRs are distinguished by FIP-related tasks, then task congruence (versus orthogonality) ought to induce differing neural responses to the same stimuli during FPVS. Additionally, we have recently developed and validated a novel paradigm to measure implicit face *memory* under varying visual conditions in controls (Nguyenet al. 2025), with an extension to SRs cohorts underway.

### Intra-individual variation: towards a more nuanced understanding of Super-Recognizers’ abilities

Broadly speaking, our results are consistent with the hypothesis that SRs’ abilities vary greatly from one to the next. But critically, this implies that *a single SR could well exhibit substantially different levels of face perception, recognition, or identification ability*. Mayer and Ramon (2023), for example, noted that SRs excelling on 3/3 diagnostic tests (the same ones employed here; c.f. Ramon 2021) were significantly better at identifying perpetrators from CCTV footage than those ‘only’ excelling on 2/3. However, explaining the relationship between behavioral task performance and FIP within a particular SR would require a more nuanced, personalized neural profile, capable of capturing the full extent of intra-individual variability across both tasks *and* subprocesses.

The current study sought only to determine only whether SRs’ FIP-related *visual processing* abilities differed from controls’ at the group level. Adding another stratum of granular detail would require larger samples and more specific behavioral correlates than those feasibly available over the course of the experiments discussed here. Thankfully, FPVS reduces experiment run-time sufficiently to test multiple subprocesses in larger samples of SRs, providing a direction for future research: correlation of FIP neuromarkers against behavioral performance to elucidate the individual differences potentially responsible for SRs’ behavioral advantages.

Finally, we certainly cannot claim to have tested FIP subprocesses *exhaustively*, so it remains possible that yet other intermediate/perceptual processes (not tested here) could explain SRs’ behavioral advantages. For instance, Hendel et al., (2019) recently found that individuals with self-reported exceptional face identity processing and high face recognition performance also showed enhanced object and word recognition. Similarly, Schroeger et al. (2023) found modest, but significantly higher contrast sensitivity among individuals with high versus low CFMT + scores. While this corroborates the hypothesis that broad variation in visual perceptual abilities could account for at least some of SRs’ reported FIP advantages, it does not exclude the possibility of task-specificity, discussed earlier. Rather, we propose that FIP neuromarkers predict such intra-individual differences in task performance, even if they do not generally distinguish SRs from controls at the group level.

At present, it is commonplace in translational research and personnel selection to employ behavioral test batteries aggregating across multiple FIP components. Yet, the available evidence—in addition to the presently discussed results—suggest that this over-simplifies the selection process, while also overlooking informative neural and behavioral individual differences. Instead employing bespoke, multi-dimensional assessments of FIP, allows for parsing variation between individuals more finely, to predict more specific behavioral abilities (Nador et al. 2022, 2024; Ramon and Rjosk 2022; Ramon and Vowels, 2025). Of course, the low prevalence of SRs in the population complicates this issue, but misusing such a scarce human resource can only lead to greater complications in practice.

## Data Availability

All data will be made available upon publication.

## Footnotes

1 Half of the SRs reported by Faghel-Soubeyrand et al. (2024), i.e. those identified in the AFC Lab, were identified in accordance with Ramon’s (2021) criteria. Detailed information on the other SRs in terms of their sub-process dependent ability is lacking, as Faghel-Soubeyrand et al. (2024) report only participants’ CFMT + scores. Similarly to the approach opted for in Bobak and collaborators’ studies, SRs reported neurophysiologically (Experiment 2) by Belanova et al. (2018) were identified using the CFMT + . Even if the usage of CFMT + is a commonality, note, however, that different authors use(d) different cut-off scores.

2 As we understand Belanova et al.’s (2018) methodological descriptions, each observer was presented 40 learning trials, based on which the authors derived “P1/N170 to measure early perceptual components”. Additionally, each observer was presented 80 old/new recognition trials, from which “only correct responses (Hits, CRs)” were considered to derive “all four ERPs”. That is, a theoretical maximum of 40 trials, given perfect old/new recognition performance, was available for before artifact and response-related rejection. Authors stated that “only participants with ≥ 15 trials in each condition were retained … to ensure reasonable signal-to-noise ratio”. Faghel-Soubeyrand et al. (2024) report having analyzed a total of “105,600 one-back trials” across all 33 observers, with no information regarding artefact rejection or minimum number of trials considered per observer was provided.

3 Moreover, with increased cognitive demands, such processes are more likely to have an impact. For instance, attention/fatigue may affect FIP performance differently across tasks probing purely perceptual processing, relative to those that pose additional requirements on memory.

## References

[CR1] Abudarham N, Bate S, Duchaine B, Yovel G (2021) Developmental prosopagnosics and super recognizers rely on the same facial features used by individuals with normal face recognition abilities for face identification. Neuropsychologia 160:107963 34284039 10.1016/j.neuropsychologia.2021.107963

[CR2] Anaki D, Kaufman Y, Freedman M, Moscovitch M (2007) Associative (prosop)agnosia without (apparent) perceptual deficits: a case-study. Neuropsychologia 45(8):1658–1671. 10.1016/j.neuropsychologia.2007.01.003 17320120 10.1016/j.neuropsychologia.2007.01.003

[CR3] Barton JJS, Davies-Thompson J, Corrow SL (2021) Prosopagnosia and disorders of face processing. Handb Clin Neurol 178:175–193. 10.1016/B978-0-12-821377-3.00006-4 33832676 10.1016/B978-0-12-821377-3.00006-4

[CR4] Bate S, Bennetts RJ, Tree JJ, Adams A, Murray E (2019) The domain-specificity of face matching impairments in 40 cases of developmental prosopagnosia. Cognition 192:104031 31351346 10.1016/j.cognition.2019.104031

[CR5] Bate S, Portch E, Mestry N (2021) When two fields collide: Identifying “super-recognisers” for neuropsychological and forensic face recognition research. Q J Exp Psychol 74(12):2154–2164 10.1177/17470218211027695 PMC8531948 34110226

[CR6] Belanova E, Davis JP, Thompson T (2018) Cognitive and neural markers of super-recognisers’ face processing superiority and enhanced cross-age effect. Cortex 108:92–111 30149236 10.1016/j.cortex.2018.07.008

[CR7] Besson G, Barragan-Jason G, Thorpe SJ, Fabre-Thorpe M, Puma S, Ceccaldi M, Barbeau EJ (2017) From face processing to face recognition: comparing three different processing levels. Cognition 158:33–43. 10.1016/j.cognition.2016.10.004 27776224 10.1016/j.cognition.2016.10.004

[CR8] Bobak AK, Bennetts RJ, Parris BA, Jansari A, Bate S (2016) An in-depth cognitive examination of individuals with superior face recognition skills. Cortex 82:48–62. 10.1016/j.cortex.2016.05.003 27344238 10.1016/j.cortex.2016.05.003

[CR9] Bobak AK, Jones AL, Hilker Z, Mestry N, Bate S, Hancock PJB (2023) Data-driven studies in face identity processing rely on the quality of the tests and data sets. Cortex 166:348–364. 10.1016/j.cortex.2023.05.018 37481857 10.1016/j.cortex.2023.05.018

[CR10] Bruck M, Cavanagh P, Ceci SJ (1991) Fortysomething: recognizing faces at one’s 25th reunion. Mem Cognit 19(3):221–228 1861608 10.3758/bf03211146

[CR12] Busigny T, Graf M, Mayer E, Rossion B (2010a) Acquired prosopagnosia as a face-specific disorder: ruling out the general visual similarity account. Neuropsychologia 48(7):2051–2067. 10.1016/j.neuropsychologia.2010.03.026 20362595 10.1016/j.neuropsychologia.2010.03.026

[CR13] Busigny T, Joubert S, Felician O, Ceccaldi M, Rossion B (2010b) Holistic perception of the individual face is specific and necessary: evidence from an extensive case study of acquired prosopagnosia. Neuropsychologia 48(14):4057–4092. 10.1016/j.neuropsychologia.2010.09.017 20875437 10.1016/j.neuropsychologia.2010.09.017

[CR14] Busigny T, Van Belle G, Jemel B, Hosein A, Joubert S, Rossion B (2014) Face-specific impairment in holistic perception following focal lesion of the right anterior temporal lobe. Neuropsychologia 56:312–333. 10.1016/j.neuropsychologia.2014.01.018 24503392 10.1016/j.neuropsychologia.2014.01.018

[CR15] Crouzet SM, Thorpe SJ (2011) Low-level cues and ultra-fast face detection. Front Psychol 2:342. 10.3389/fpsyg.2011.00342 22125544 10.3389/fpsyg.2011.00342 PMC3221302

[CR16] Crouzet SM, Kirchner H, Thorpe SJ (2010) Fast saccades toward faces: face detection in just 100 ms. J vis 10(4):16.1-17. 10.1167/10.4.16 20465335 10.1167/10.4.16

[CR17] Davies-Thompson J, Pancaroglu R, Barton J (2014) Acquired prosopagnosia: structural basis and processing impairments. Front Biosci (Elite Ed) 6(1):159–174. 10.2741/e699 24389150 10.2741/e699

[CR18] De Renzi E, Faglioni P, Grossi D, Nichelli P (1991) Apperceptive and associative forms of prosopagnosia. Cortex 27(2):213–221. 10.1016/s0010-9452(13)80125-6 1879150 10.1016/s0010-9452(13)80125-6

[CR19] Delvenne J-F, Seron X, Coyette F, Rossion B (2004) Evidence for perceptual deficits in associative visual (prosop)agnosia: a single-case study. Neuropsychologia 42(5):597–612. 10.1016/j.neuropsychologia.2003.10.008 14725798 10.1016/j.neuropsychologia.2003.10.008

[CR20] Dunn JD, Summersby S, Towler A, Davis JP, White D (2020) UNSW face test: a screening tool for Super-Recognizers. PLoS ONE 15(11):e0241747. 10.1371/journal.pone.0241747 33196639 10.1371/journal.pone.0241747 PMC7668578

[CR22] Faghel-Soubeyrand S, Ramon M, Bamps E, Zoia M, Woodhams J, Richoz AR, Caldara R, Gosselin F, Charest I (2024) Decoding face recognition abilities in the human brain. PNAS Nexus 3(3):pgae095. 10.1093/pnasnexus/pgae095 38516275 10.1093/pnasnexus/pgae095 PMC10957238

[CR23] Földiák P, Xiao D, Keysers C, Edwards R, Perrett DI (2004) Rapid serial visual presentation for the determination of neural selectivity in area STSa. Prog Brain Res 144:107–116 14650843 10.1016/s0079-6123(03)14407-x

[CR24] Fysh MC, Stacchi L, Ramon M (2020) Differences between and within individuals, and subprocesses of face cognition: implications for theory, research and personnel selection. R Soc Open Sci 7(9):200233. 10.1098/rsos.200233 33047013 10.1098/rsos.200233 PMC7540753

[CR103] Gratton G, Coles MG, Donchin E (1983) A new method for off-line removal of ocular artifact.Electroencephalogr Clin Neurophysiol. 55(4):468–84. 10.1016/0013-4694(83)90135-9 6187540 10.1016/0013-4694(83)90135-9

[CR104] Hendel RK, Starrfelt R, Gerlach C (2019) The good, the bad, and the average: characterizing therelationship between face and object processing across the face recognition spectrum.Neuropsychologia 124:274–284. 10.1016/j.neuropsychologia.2018.11.016 30529245 10.1016/j.neuropsychologia.2018.11.016

[CR25] Jacques C, Retter TL, Rossion B (2016) A single glance at natural face images generate larger and qualitatively different category-selective spatio-temporal signatures than other ecologically-relevant categories in the human brain. Neuroimage 137:21–33 27138205 10.1016/j.neuroimage.2016.04.045

[CR26] Jacques C, Rossion B, Volfart A, Brissart H, Colnat-Coulbois S, Maillard L, Jonas J (2020) The neural basis of rapid unfamiliar face individuation with human intracerebral recordings. Neuroimage 221:117174. 10.1016/j.neuroimage.2020.117174 32682990 10.1016/j.neuroimage.2020.117174

[CR27] Jenkins R, White D, Van Montfort X, Burton AM (2011) Variability in photos of the same face. Cognition 121(3):313–323 21890124 10.1016/j.cognition.2011.08.001

[CR102] Linka M, Broda MD, Alsheimer T, de Haas B, Ramon M (2022) Characteristic fixation biases in Super-Recognizers. J Vis 22(8):17.10.1167/jov.22.8.17 35900724 10.1167/jov.22.8.17 PMC9344214

[CR28] Liu J, Harris A, Kanwisher N (2002) Stages of processing in face perception: an MEG study. Nat Neurosci 5(9):910–916. 10.1038/nn909 12195430 10.1038/nn909

[CR29] Liu-Shuang J, Torfs K, Rossion B (2016) An objective electrophysiological marker of face individualisation impairment in acquired prosopagnosia with fast periodic visual stimulation. Neuropsychologia 83:100–113. 10.1162/jocn_a_01126 26318239 10.1016/j.neuropsychologia.2015.08.023

[CR100] Marini F, Manassi M, Ramon M (2024) Super recognizers: increased sensitivity or reduced biases? Insights from serial dependence. J Vis 24(7):13. 10.1167/jov.24.7.13 39046722 10.1167/jov.24.7.13 PMC11271810

[CR30] Mayer M, Ramon M (2023) Improving forensic perpetrator identification with Super-Recognizers. Proc Natl Acad Sci U S A 120(20):e2220580120. 10.1073/pnas.2220580120 37159477 10.1073/pnas.2220580120 PMC10193965

[CR32] Nador JD, Ramon M (2022) Fast periodic visual stimulation reveals expedited neural face processing in Super-Recognizers. J vis 22(14):4163. 10.1167/jov.22.14.4163

[CR35] Nador JD, Alsheimer TA, Gay A, Ramon M (2021a) Image or identity? Only Super-Recognizers’ (memor)ability is consistently viewpoint-invariant. Swiss Psychology Open 1. https://osf.io/preprints/psyarxiv/jgvuk/download

[CR33] Nador JD, Zoia M, Pachai MV, Ramon M (2021b) Psychophysical profiles in Super-Recognizers. Sci Rep 11(1):13184 34162959 10.1038/s41598-021-92549-6 PMC8222339

[CR34] Nador JD, Vomland M, Thielgen MM, Ramon M (2022) Face recognition in police officers: who fits the bill? Forensic Sci Int Rep 5:100267. 10.1016/j.fsir.2022.100267

[CR36] Nador JD, Uittenhove K, Ramon M (2024) Super-Recognizers or su-perceivers. In: Talk presented at European conference on visual perception

[CR37] Nguyen T, Ficco L, Thornton I, Ramon M (2025) An ecological and objective neural marker of implicit identity recognition. In: Presentation at the 8^th^ cognitive computational neuroscience conference

[CR38] Phillips PJ, Yates AN, Hu Y, Hahn CA, Noyes E, Jackson K, Cavazos JG, Jeckeln G, Ranjan R, Sankaranarayanan S, Chen J-C, Castillo CD, Chellappa R, White D, O’Toole AJ (2018) Face recognition accuracy of forensic examiners, superrecognizers, and face recognition algorithms. Proc Natl Acad Sci U S A 115(24):6171–6176 29844174 10.1073/pnas.1721355115 PMC6004481

[CR39] Ramon M (2021) Super-Recognizers–a novel diagnostic framework, 70 cases, and guidelines for future work. Neuropsychologia 158:107809. 10.1016/j.neuropsychologia.2021.107809 33662395 10.1016/j.neuropsychologia.2021.107809

[CR101] Ramon M (2023) Unique traits, computational insights: studying Super-Recognizers for societal applications. 10.31234/osf.io/8zejy

[CR40] Ramon M, Gobbini MI (2018) Familiarity matters: a review on prioritized processing of personally familiar faces. Vis Cogn 26(3):179–195. 10.1080/13506285.2017.1405134

[CR45] Ramon M, Rjosk S (2021) Super-Recognizers in policing–best practices established during development of the Berlin Model for SR-identification. In: European Recommendations for the Protection of Public Spaces against Terrorist Attacks (Polizei Berlin, 2021). https://joint-research-centre.ec.europa.eu/scientific-activities-z/protection-public-spaces-terrorist-attacks_en

[CR46] Ramon M, Rjosk S (2022) beSure – Berlin test for super-recognizer identification: Part I: development. Verlag für Polizeiwissenschaft, Prof. Dr. Clemens Lorei

[CR47] Ramon M, Vowels MJ (2025) Demonstrating the Super-Recognizer advantage for law enforcement. 10.31234/osf.io/x6ryw_v2 10.1016/j.isci.2026.116456 PMC13378012 42491494

[CR41] Ramon M, Busigny T, Rossion B (2010) Impaired holistic processing of unfamiliar individual faces in acquired prosopagnosia. Neuropsychologia 48(4):933–944. 10.1016/j.neuropsychologia.2009.11.014 19944710 10.1016/j.neuropsychologia.2009.11.014

[CR42] Ramon M, Bobak AK, White D (2019a) Super-Recognizers: from the lab to the world and back again. Br J Psychol 110(3):461–479. 10.1111/bjop.12368 30893478 10.1111/bjop.12368 PMC6767378

[CR43] Ramon M, Bobak AK, White D (2019b) Towards a ‘manifesto’ for Super-Recognizer research. Br J Psychol 110(3):495–498. 10.1111/bjop.12411 31231789 10.1111/bjop.12411 PMC6771599

[CR44] Ramon M, Vowels M, Groh M (2024) Deepfake detection in Super-Recognizers and police officers. IEEE Secur Priv 22:68–76. 10.1109/MSEC.2024.3371030

[CR48] Retter TL, Jiang F, Webster MA, Rossion B (2018) Dissociable effects of inter-stimulus interval and presentation duration on rapid face categorization. Vis Res 145:11–20 29581059 10.1016/j.visres.2018.02.009 PMC6485415

[CR49] Retter TL, Jiang F, Webster MA, Rossion B (2020) All-or-none face categorization in the human brain. Neuroimage 213:116685 32119982 10.1016/j.neuroimage.2020.116685 PMC7339021

[CR50] Retter TL, Jiang F, Webster MA, Michel C, Schiltz C, Rossion B (2021) Varying stimulus duration reveals consistent neural activity and behavior for human face individuation. Neuroscience 472:138–156 34333061 10.1016/j.neuroscience.2021.07.025

[CR51] Robertson DJ, Noyes E, Dowsett AJ, Jenkins R, Burton AM (2016) Face recognition by metropolitan police super-recognisers. PLoS ONE 11(2):e0150036 26918457 10.1371/journal.pone.0150036 PMC4769018

[CR52] Rossion B (2014) Understanding face perception by means of prosopagnosia and neuroimaging. Front Biosci (Elite Ed) 6(2):258–307. 10.2741/E706 24896206 10.2741/E706

[CR53] Rossion B (2018) Damasio’s error—prosopagnosia with intact within-category object recognition. J Neuropsychol 12(3):357–388. 10.1111/jnp.12162 29845731 10.1111/jnp.12162

[CR54] Rossion B, Caharel S (2011) ERP evidence for the speed of face categorization in the human brain: disentangling the contribution of low-level visual cues from face perception. Vis Res 51(12):1297–1311. 10.1016/j.visres.2011.04.003 21549144 10.1016/j.visres.2011.04.003

[CR55] Rossion B, Torfs K, Jacques C, Liu-Shuang J (2015) Fast periodic presentation of natural images reveals a robust face-selective electrophysiological response in the human brain. J vis 15(1):18–18 10.1167/15.1.18 25597037

[CR56] Rossion B, Jacques C, Jonas J (2018) Mapping face categorization in the human ventral occipitotemporal cortex with direct neural intracranial recordings. Ann N Y Acad Sci. 10.1111/nyas.13596 29479704 10.1111/nyas.13596

[CR57] Rossion B, Retter TL, Liu-Shuang J (2020) Understanding human individuation of unfamiliar faces with oddball fast periodic visual stimulation and electroencephalography. Eur J Neurosci 52(10):4283–4344. 10.1111/ejn.14865 32542962 10.1111/ejn.14865

[CR58] Russell R, Duchaine B, Nakayama K (2009) Super-Recognizers: people with extraordinary face recognition ability. Psychon Bull Rev 16(2):252–257. 10.3758/PBR.16.2.252 19293090 10.3758/PBR.16.2.252 PMC3904192

[CR59] Schroeger A, Ficco L, Wuttke SJ, Kaufmann JM, Schweinberger SR (2023) Differences between high and low performers in face recognition in electrophysiological correlates of face familiarity and distance-to-norm. Biol Psychol 182:108654. 10.1016/j.biopsycho.2023.108654 37549807 10.1016/j.biopsycho.2023.108654

[CR60] Stacchi L, Liu-Shuang J, Ramon M, Caldara R (2019a) Reliability of individual differences in neural face identity discrimination. Neuroimage 189:468–475 30654176 10.1016/j.neuroimage.2019.01.023

[CR61] Stacchi L, Ramon M, Lao J, Caldara R (2019b) Neural representations of faces are tuned to eye movements. J Neurosci 39(21):4113–4123 30867260 10.1523/JNEUROSCI.2968-18.2019 PMC6529871

[CR62] Stacchi L, Huguenin-Elie E, Caldara R, Ramon M (2020) Normative data for two challenging tests of face matching under ecological conditions. Cogn Res Princ Implic 5:1–17 32076893 10.1186/s41235-019-0205-0 PMC7031457

[CR64] Thorpe S, Fize D, Marlot C (1996) Speed of processing in the human visual system. Nature 381(6582):520–522 8632824 10.1038/381520a0

[CR65] Tsao DY, Livingstone MS (2008) Mechanisms of face perception. Annu Rev Neurosci 31:411–437. 10.1146/annurev.neuro.30.051606.094238 18558862 10.1146/annurev.neuro.30.051606.094238 PMC2629401

[CR66] Wang P, Nikolić D (2011) An LCD monitor with sufficiently precise timing for research in vision. Front Hum Neurosci 5:85 21887142 10.3389/fnhum.2011.00085 PMC3157744

[CR68] Xu B, Liu-Shuang J, Rossion B, Tanaka J (2017) Individual differences in face identity processing with fast periodic visual stimulation. J Cogn Neurosci 29(8):1368–1377 28358660 10.1162/jocn_a_01126

[CR69] Yovel G, Levy J, Grabowecky M, Paller KA (2003) Neural correlates of the left-visual-field superiority in face perception appear at multiple stages of face processing. J Cogn Neurosci 15(3):462–474. 10.1162/089892903321593162 12729496 10.1162/089892903321593162

